# Engineered probiotic *Escherichia coli* can eliminate and prevent *Pseudomonas aeruginosa* gut infection in animal models

**DOI:** 10.1038/ncomms15028

**Published:** 2017-04-11

**Authors:** In Young Hwang, Elvin Koh, Adison Wong, John C. March, William E. Bentley, Yung Seng Lee, Matthew Wook Chang

**Affiliations:** 1 Department of Biochemistry, Yong Loo Lin School of Medicine, National University of Singapore, Singapore 117596, Singapore; 2 NUS Synthetic Biology for Clinical and Technological Innovation (SynCTI), Life Sciences Institute, National University of Singapore, Singapore 117456, Singapore; 3 Department of Biological and Environmental Engineering, Cornell University, Ithaca, New York 14853, USA; 4 Fischell Department of Bioengineering, University of Maryland, College Park, Maryland 20742, USA; 5 Department of Paediatrics, Yong Loo Lin School of Medicine, National University of Singapore, Singapore S 119074, Singapore

## Abstract

Bacteria can be genetically engineered to kill specific pathogens or inhibit their virulence. We previously developed a synthetic genetic system that allows a laboratory strain of *Escherichia coli* to sense and kill *Pseudomonas aeruginosa in vitro*. Here, we generate a modified version of the system, including a gene encoding an anti-biofilm enzyme, and use the probiotic strain *Escherichia coli* Nissle 1917 as host. The engineered probiotic shows *in vivo* prophylactic and therapeutic activity against *P. aeruginosa* during gut infection in two animal models (*Caenorhabditis elegans* and mice). These findings support the further development of engineered microorganisms with potential prophylactic and therapeutic activities against gut infections.

## Abstract (web summary)

Bacteria can be engineered to kill specific pathogens. Here, the authors modify and optimize a synthetic genetic system in a probiotic strain of *Escherichia coli*, and show that the engineered probiotic can sense and kill the pathogen *Pseudomonas aeruginosa* in two animal models of gut infection.

Bacterial infections are a major cause of morbidity and mortality in clinical settings; their impact is exacerbated by the epidemic-like emergence of resistant strains^1^. In addition, antibiotic therapies can eliminate beneficial microbes and lead to the persistence of resistant strains. With growing interest in the importance of the regulatory roles played by microbiota in human health, studies are revealing potential means by which native microbial communities can be exploited to our advantage, thereby providing strategic approaches for managing infections. On the basis of advances in genome engineering and on our understanding of pathogenesis, a microbe-based pathogen-specific antimicrobial strategy that detects and targets infectious pathogens with precision can be envisioned.

Probiotics, such as *Escherichia coli* Nissle 1917, are microorganisms that confer beneficial physiological or therapeutic activities. For example, some probiotics can alleviate intestinal inflammation and irritable bowel syndrome, strengthen host innate immune functions or protect the intestinal barrier against pathogens^2^,^3^,^4^. Further, probiotics can prevent colonization of the human intestinal tract by pathogenic *E. coli*^5^,^6^. A few studies have exploited engineered probiotics for therapeutic purposes, including site-specific expression and delivery of biomolecules that target bacterial and viral infections^7^,^8^,^9^,^10^,^11^ or as adjuvants to pharmacological therapy^12^. However, the majority of such studies have been validated *in vitro*, and only a few have been evaluated for therapeutic efficiency using *in vivo* models^13^,^14^,^15^,^16^.

Here, we evaluate the efficacy of an engineered probiotic strain against *Pseudomonas aeruginosa* infection in two *in vivo* infection models. Although the lungs are a major site of infection by this opportunistic pathogen in immune-deficient patients^17^,^18^,^19^, the intestinal tract is considered to be an important reservoir of *P. aeruginosa*^20^ for opportunistic infections in neutropenic and immunocompromised patients^21^,^22^,^23^, and the presence of this pathogen in the intestinal tract facilitates hematogenous spread to the lungs^24^. In addition to the initiation of respiratory infections from the GI tract, *P. aeruginosa* gut infection is also responsible for increased mortality in gut-derived sepsis and bacteremia^25^,^26^ and causes inflammatory bowel diseases including typhlitis and Shanghai fever^27^. Furthermore, colonization of the GI tract by *P. aeruginosa* in low-birth-weight infants leads to severe necrotizing enterocolitis and a significant increase in mortality^28^.

We previously showed that an engineered microbe can be reprogrammed to detect a specific pathogen (*P. aeruginosa*) by detection of its secreted autoinducer N-acyl homoserine lactone (AHL) and to respond by inducing its own lysis (driven by lysin E7), thus releasing an anti-*P. aeruginosa* toxin, pyocin S5. The study demonstrated efficient pathogen-specific killing activity in *in vitro* co-cultures^29^. Here, in addition, we have employed an anti-biofilm enzyme, dispersin B (DspB), to promote the destabilization of mature biofilms to achieve a more apt and physiologically relevant treatment. DspB is naturally produced by *Actinobacillus actinomycetemcomitans* and hydrolyses 1,6-*N*-acetyl-D-glucosamine, which is known to stabilize biofilm formation and increase biofilm integrity^30^.

Plasmids provide a common framework for synthetic circuits. They are typically retained under the pressure of an antibiotic selection marker, but this presents a potential risk for horizontal gene transfer of the antibiotic resistance to other bacteria. To address this issue, we introduced an auxotrophic marker in *E. coli* Nissle; the vector-host combination consists of an *E. coli* Nissle Δ*alr* Δ*dadX* strain that requires D-alanine for growth and a plasmid providing the wild-type copy of *alr* in *trans* to complement the deletion. The *dadX* and *alr* genes encode two alanine racemases that catalyse the interconversion of D-alanine and L-alanine; the D-enantiomer is used in the construction of the peptidoglycan layer of bacterial cell walls.

We tested the system *in vivo* using *Caenorhabditis elegans* and murine infection models. In both systems, the engineered probiotic *E. coli* Nissle autonomously executes diagnostic and therapeutic activities that efficiently reduce a pre-colonized *P. aeruginosa* infection. Furthermore, the engineered probiotic strain exhibited prophylactic activity by conferring specific protection against *P. aeruginosa* infection.

## Results

### Engineering *E. coli* Nissle for optimal *in vivo* expression

**Figure 1:**
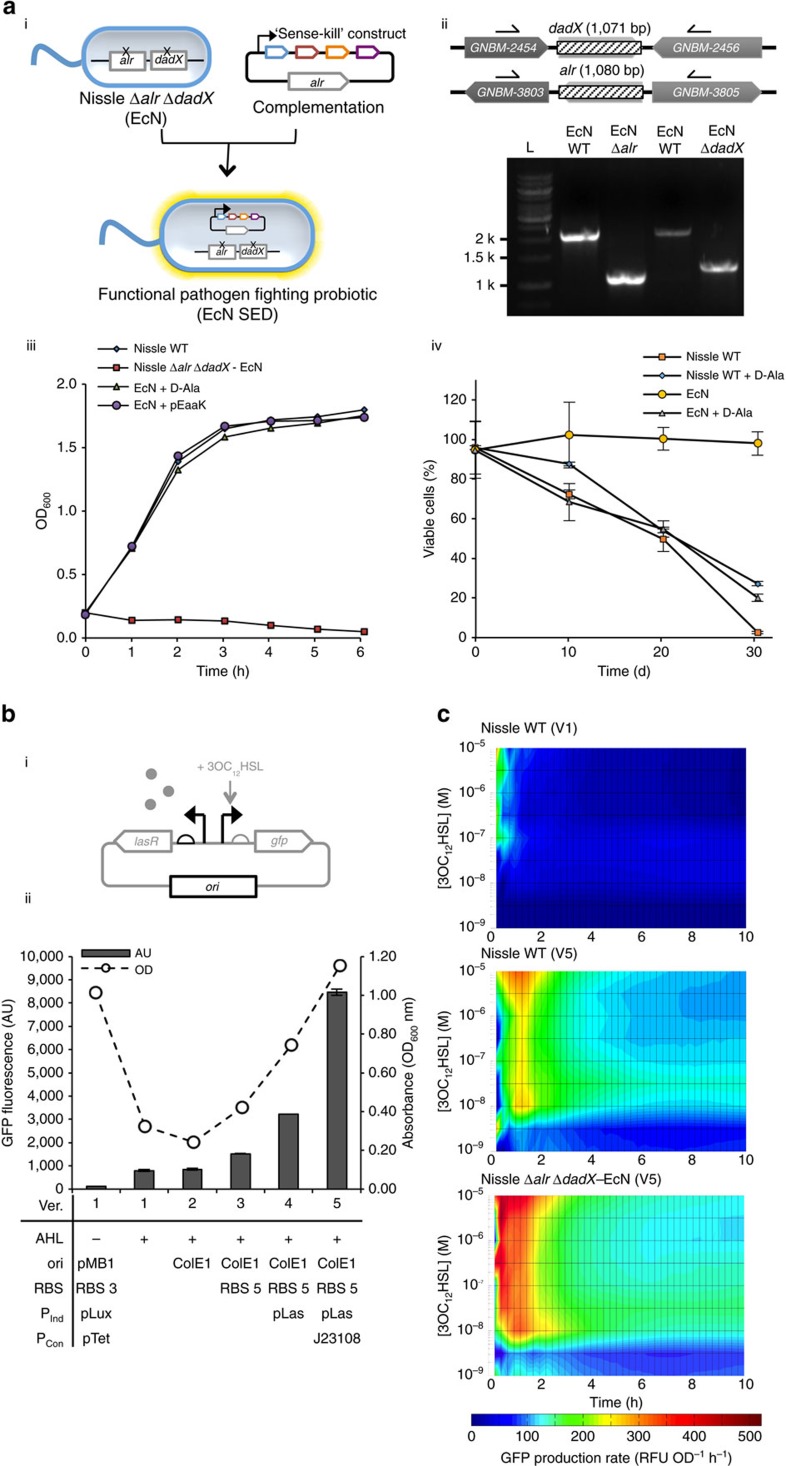
Development of optimal cellular host and genetic element systems for a functional probiotic strain. (**a**) (i) Vector-host system created in the *E. coli* Nissle strain by auxotrophic complementation to stabilize plasmid retention. The ‘Sense-Kill' construct comprises of: lasR (blue), pyocin S5 (brown), E7 lysis protein (orange) and DspB (purple) (Supplementary Table 1). (ii) The chromosomal deletion of *alr* and *dadX* in *E. coli* Nissle was confirmed by PCR. Genes flanking the deleted region are indicated by their annotated gene names: *GNBM-2454* (D-amino acid dehydrogenase small subunit), *GNBM-2456* (cell volume regulation protein A), *GNBM-3803* (ribosyl nicotinamide transporter, PnuC-like), *GNBM-3805* (hypothetical protein). (iii) Growth of strain EcN (Nissle Δ*alr* Δ*dadX*) in the presence of exogenous D-alanine (50 μg ml^−1^) or in the presence of complementary vector (pEaaK; Supplementary Fig. 1). The microtiter plate assay was performed in triplicate wells; the mean and s.e.m. (error bars) from three experiments are shown. (iv) Plasmid retention was tested by culturing wild-type Nissle (non-auxotrophic) and EcN (auxotrophic) cells containing plasmid pEaaK (which includes a kanamycin resistance gene) in the absence of antibiotic and in the presence or absence of exogenous D-alanine in minimal medium. Cell viability in the presence of kanamycin was used to infer plasmid retention and expression. The mean and s.e.m. (error bars) from three experiments are shown. (**b**) (**i**) Optimization of the regulatory components of the sensing system (highlighted in black in the circuit diagram) was performed. (ii) The effects of several variations in the regulatory components on GFP expression and bacterial growth were assessed. The mean and s.e.m. (error bars) from three experiments are shown. (**c**) GFP production per cell over time at different 3OC_12_-HSL inducer concentrations in Nissle and EcN cells containing the reporter construct. The optimized circuit (V5) showed a lower threshold for 3OC_12_-HSL detection and sustained expression in the new vector-host system.

In *E. coli* Nissle, the *alr* and *dadX* genes encode alanine racemases, which are essential enzymes in D-alanine metabolism^31^,^32^. D-Alanine is an essential building block of Gram-negative peptidoglycan; when both the *alr* and *dadX* genes are inactivated, cells become dependent on exogenous D-alanine for survival^31^. We exploited this obligate requirement for alanine racemase to develop an *alr*-dependent vector-host system in *E. coli* Nissle (Fig. 1a). We introduced gene deletions using a previously described method^33^; the resulting Nissle Δ*alr* Δ*dadX* strain (referred to as EcN hereafter) required D-alanine for growth. For complementation, an alr+ plasmid (pEaaK) was constructed by introducing the native *alr* gene promoter and Shine–Dalgarno (SD) sequences from wild-type Nissle into the BglBrick backbone^34^ (Supplementary Fig. 1A). EcN complemented with pEaaK grew in the absence of D-alanine, and its growth rate and viability were comparable to that of wild-type Nissle (Fig. 1a). Furthermore, protein expression and plasmid retention in antibiotic-free medium was greatly improved in the pEaaK-containing Nissle (EcN) compared to wild-type Nissle (Fig. 1a; Supplementary Fig. 1D).

Due to the ColE1 *ori* used for pEaaK, the level of alanine racemase expressed might cause an additional metabolic burden^35^,^36^,^37^ or potentially affect cell wall synthesis. The expression of *alr* did not result in a discernible deficit in cell growth, nor did it impede cell-lysis mediated by the E7 protein (Supplementary Fig. 1B). Interestingly, it was further noted that the expression of exogenous alanine racemase by our *alr*+ plasmid resulted in a slightly higher growth rate at low pH comparable to that found in a gastric environment (Supplementary Fig. 1C). As supplementation of cultures of wild-type Nissle with excess D-alanine also resulted in an improved growth rate, it is postulated that the phenomenon is a result of a higher level of alanine racemase expression compared to the wild-type.

We reconfigured this new Nissle strain in our previously described ‘Sense-Kill' genetic system^29^. It was noted that considerable reduction in GFP expression of the sensing genetic system occurred when the *alr*+ plasmid containing previous GFP reporter system was introduced to wild-type *E. coli* Nissle (Fig. 1b,c). As the recombinant protein expression shows a certain strain-context dependence, genetic components regulating expression in response to the quorum sensing molecule, 3OC_12_HSL, have been systematically optimized for expression in EcN (D-alanine auxotrophic *E. coli* Nissle) and incorporated into our alr+ vector (Fig. 1b, Supplementary Fig. 2). When the cells were induced with QS molecules and the rates of expression and cell growth were observed over time, the optimized system resulted in a lower threshold of 3OC_12_HSL detection (from 1 μM to 10 nM) and prolonged robust expression of alanine racemase upon induction (Fig. 1c). The heat map shows the rate of expression (Fluorescence/OD_600nm_/time); the optimized system (V5) was found to produce a more rapid response and a higher rate of GFP expression upon induction than the other systems. The rate of production persisted for ∼2 h, followed by an extended period of lower expression. It is possible that the observed reduction in expression after 2 h is caused by the reallocation of cellular resources to favour cellular growth and essential protein expression over orthogonal expression^35^,^36^,^37^.

### Engineered *E. coli* Nissle against various stages of *P. aeruginosa*

**Figure 2:**
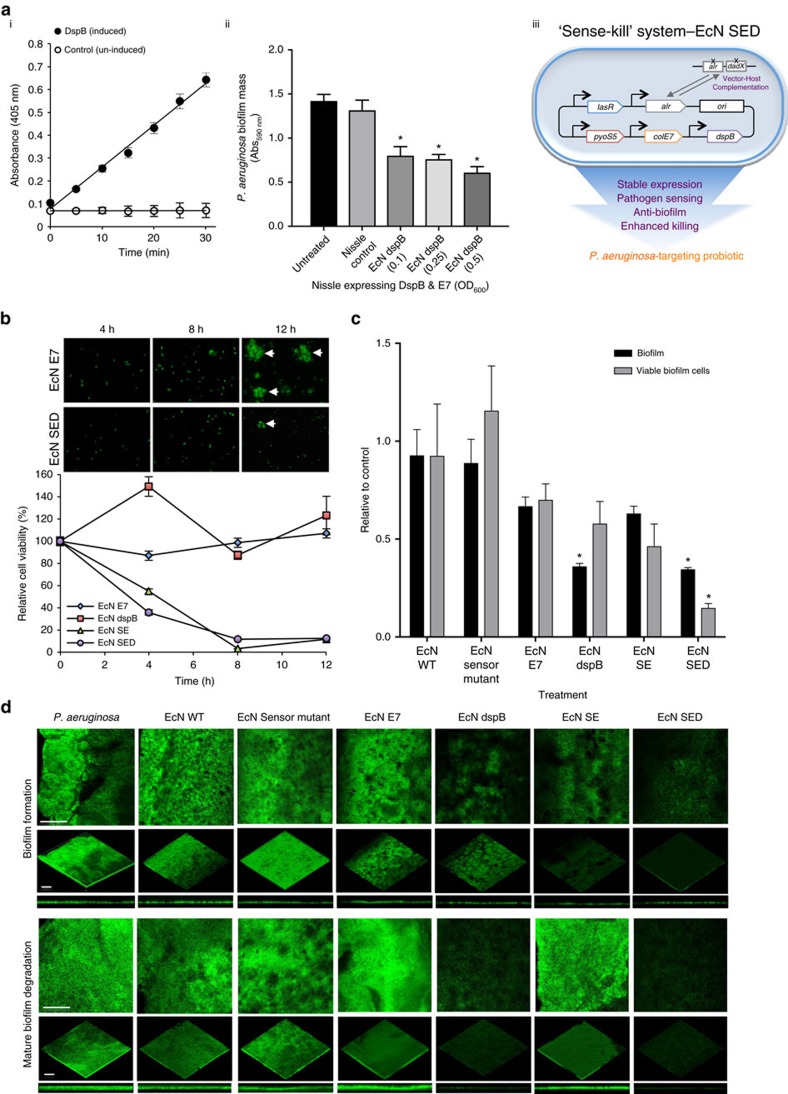
*In vitro* functional assays of the engineered probiotic strain. (**a**) (i) Glycosyl hydrolase activity of DspB with 4-nitrophenyl-N-acetyl-β-D-glucosaminide as substrate. The optical density at 405 nm (OD_405 nm_) is proportional to the amount of 4-nitrophenolate released in the reaction. (ii) Anti-biofilm activity of DspB expressed by the varying number of EcN cells (OD_600 nm_) against mature *P. aeruginosa* biofilm. EcN cells expressing DspB & E7 under quorum sensor are termed ‘EcN dspB'; the change in biofilm biomass was quantified by crystal violet staining. ‘Untreated' refers to mature *P. aeruginosa* biofilm. The Nissle control is *P. aeruginosa* biofilm treated with wild-type EcN cells. The mean and s.e.m. (error bars) from three experiments are shown. * indicates statistical significance (Student *t*-test, *P*<0.05) compared to Untreated and to the Nissle control. (iii) Schematic representation of engineered EcN with the new ‘Sense-kill' killing circuit, EcN SED (S5 pyocin, E7 lysis protein and Dispersin B), in the auxotrophic vector-host complementation system to produce an optimized *P. aeruginosa*-targeting probiotic. (**b**) *P. aeruginosa* viability at the indicated time points of co-culture with EcN SED in comparison with lysis control (E7), EcN dspB and EcN expressing S5 & E7 (EcN SE). The data are presented as relative cell survival of *P. aeruginosa* cells in each treatment group compared to the *P. aeruginosa* control group at each time point. Small aliquots of each co-culture were taken at the indicated time points for microscopic observation. The arrow indicates formation of microcolonies. (**c**) Anti-biofilm activity was measured by quantifying biofilm mass (by crystal violet staining) and viable biofilm cells (by cell viability counting) after treating mature biofilm with engineered EcN cells for 6 h. The results are shown relative to the *P. aeruginosa* control groups. The mean and s.e.m. (error bars) from three experiments are shown. * indicates statistical significance at an alpha value of <0.05 by one-way ANOVA with the Bonferroni multiple comparisons test. (**d**) *P. aeruginosa* biofilm containing the GFP expression vector was grown on 8-well chambered glass slides for 48 h; the biofilm was subsequently treated with the engineered EcN for 6 h and visualized by confocal laser scanning microscopy (CLSM). *P. aeruginosa* was co-cultured with engineered EcN for 20 h to evaluate its effect on biofilm formation. Images were reconstructed from biofilm Z-stacks (30-μm thickness) using ImageJ. The scale bars represent 100 μm.

To evaluate the new strain with improved autoinducer sensitivity coupled with more robust downstream expression, the engineered EcN with anti-microbial and anti-biofilm activity was tested against *P. aeruginosa*. To add an anti-biofilm activity to the ‘Sense-Kill' construct, dispersin B (DspB) was expressed and tested for glycosyl hydrolase activity. In Fig. 2a, it can be seen that the glycosyl hydrolase activity of the cells increased linearly with time after induction. The corresponding biofilm dispersal activity is also shown. The revised killing circuit, therefore, consists of previously demonstrated *P. aeruginosa*-specific bacteriocin, the S5 pyocin and E7 lysis proteins^29^, and the new anti-biofilm enzyme, DspB, in an alanine auxotroph (denoted EcN SED); all of these are actuated in an autonomous manner in response to autoinducers secreted by *P. aeruginosa*. *P. aeruginosa* constitutively expressing GFP was co-cultured with EcN SED at various starting cell ratios based on OD_600_ values. The growth rate of the cells, as determined based on constitutive GFP expression (Supplementary Fig. 3A), shows a significant reduction at equivalent starting OD_600_ of EcN SED compared to its lysis control, EcN E7. Further viability assays confirmed the observed reduction in growth rate to be a result of efficient killing of the pathogen in this system. When converted to actual colony-forming units (Supplementary Fig. 3B), this ratio demonstrates that a significantly higher degree of inhibition was achieved at a lower ratio of engineered EcN cells to the targeted *P. aeruginosa* cells; under these conditions, a single-EcN cell was seemingly able to kill three *P. aeruginosa* cells. To provide appropriate controls for EcN SED, lysis control groups (E7 (EcN E7), S5 and E7 (EcN SE), DspB and E7 (EcN dspB)), non-lytic controls (EcN wild-type (WT) and the EcN Sensor mutant, which contains S5, E7 and DspB with inactivated *lasR*), were included in the experiments; the results of the control experiments are summarized in Supplementary Fig. 4. In preventing biofilm formation and killing *P. aeruginosa* cells, EcN SE and EcN SED show the greatest reduction in viable cells, resulting in a greatly decreased number of cells available to form microcolonies (Fig. 2b). We note, however, that EcN SE expressing S5 was unable to penetrate mature biofilms, precluding a reduction in biofilm mass or in the number of biofilm-encapsulated cells. In addition, although EcN dspB was able to degrade mature biofilms, it did not inhibit biofilm formation or reduce the number of biofilm-encapsulated cells. EcN SED, however, which expresses both S5 and DspB, was able to disassemble mature biofilm structures, resulting in the efficient killing of potentially antibiotic-resistant cells encapsulated within the biofilm matrix (an 80% reduction compared to untreated controls; Fig. 2c,d).

### Engineered *E. coli *Nissle in *P. aeruginosa*-infected *C. elegans*

**Figure 3:**
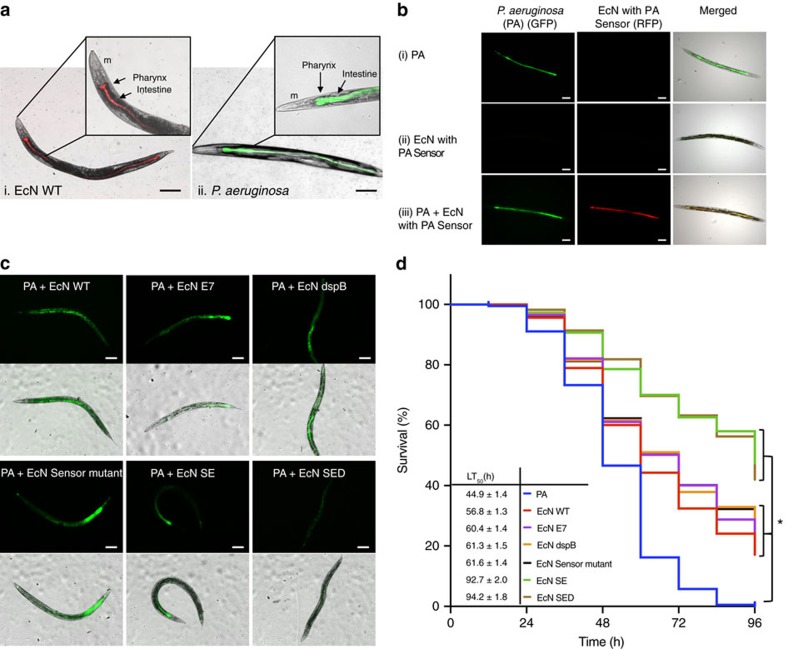
Evaluation of the engineered probiotic strain in a *C. elegans* infection model. (**a**) Fluorescence images of *C*. *elegans* show the localization of (i) wild-type Nissle expressing mRFP1; (ii) *P. aeruginosa* (PA) expressing GFP. m=mouth. The scale bars represent 100 μm. (**b**) *C. elegans* were given the following for 24 h and viewed by fluorescence microscopy: (i) *P. aeruginosa* (constitutively expressing GFP) alone; (ii) EcN expressing RFP in the presence of AHL (PA sensor device with RFP); (iii) *P*. *aeruginosa* followed by EcN with PA Sensor (for 4 h) before viewing. The scale bars represent 100 μm. (**c**) At 24 h post-infection, the nematodes were divided into the following treatment groups; untreated (PA); treated with engineered EcN SED and its control that expresses either E7 alone, S5 & E7 only (SE), DspB & E7 (dspB) or Sensor mutant (disrupted lasR with S5, E7 and dspB gene intact). Representative images of nematodes treated with all the variants of EcN are shown to demonstrate the clearance of *P. aeruginosa* (GFP). The scale bars represent 100 μm. (**d**) The survival rate of each treatment group was quantified until all the nematodes in the infection group had died (96 h). The significance of the results was determined by the Mantel–Haenszel log-rank test followed by Bonferroni's correction. Statistically significant differences in survival among the groups were not observed. The data from three independent experiments are shown (*n*=80–100). *P<0.001.

A simple infection model was established in *C. elegans* to assess the ability of the engineered EcN to treat *P. aeruginosa* infection. The nematodes were infected with *P. aeruginosa* expressing GFP before treatment with engineered EcN SED or with the EcN controls. Gut infections and nematode survival rates were monitored. The infectivity of *P. aeruginosa* was first established as observed by its accumulation in the digestive tract (Fig. 3a). Pathogen-specific activation of ingested EcN SED was further verified by constructing EcN expressing RFP under the control of a genetically encoded sensing system (Fig. 3b). Sensor-specific activation was confirmed by the lack of downstream expression when the sensor was inactivated; in this case, EcN with the sensor mutation resulted in survival comparable to that produced by treatment of *P. aeruginosa*-infected nematodes with wild-type EcN. The ability of the pathogen to kill the nematodes was measured by determining the half-maximal lethal time (the time required to kill 50% of the nematodes, LT_50_) and the survival rate. In addition, changes in the localization of *P. aeruginosa* were observed via fluorescence microscopy.

Initially, any potential effect of EcN cells on the survival of *C. elegans* was evaluated in comparison to the normal food source, *E. coli* OP50 (Supplementary Fig. 5B). It was observed that the survival of nematodes was not affected by feeding EcN cells. Next, *C. elegans* were infected with *P. aeruginosa* followed by EcN cells to assess the anti-pathogenic activity of the engineered EcN cells. Interestingly, an increase in survival was observed when infected nematodes were given any EcN cells (including the controls and all of the variants) compared to the infection control group. We note additionally that there was no clear difference in survival among the control variants of EcN. Importantly, the greatest increase in survival rate occurred in the EcN SE and EcN SED treatment groups; in these groups, the survival time increased >2-fold (LT_50_ increased from 44.9 h to 92.7–94.2 h), and ∼50% of the nematodes in the EcN SE and SED treatment groups remained alive at 96 h post-infection, at which time all the infected nematodes in the infection control group had died. The engineered EcN presumably contributed to the improved survival rate by promoting enhanced clearance of *P. aeruginosa* from the digestive tract, as the greatest extent and frequency of clearance was observed in the EcN SED group (Supplementary Fig. 5A).

It is notable that although there were differences in the survival of infected *C. elegans* treated with EcN with and without S5 pyocin, there was no discernible difference in survival or pathogen clearance between the EcN SE and EcN SED *C. elegans* treatment groups. As the activity of DspB against biofilms was clearly observed in Fig. 2, it is possible that either the *C. elegans* infection model is insufficient for evaluating chronic infection (despite our use of a slow-killing mode of infection^38^) or biofilm formation within *C. elegans* does not greatly contribute to death in *C. elegans*. To further verify the presence or absence of beneficial activity of S5 and DspB co-expression, we examined the relevant protein expression levels. EcN SED is faced with a higher metabolic burden than EcN SE because it expresses an additional protein in the face of limited cellular resources^35^,^36^,^37^. Therefore, EcN SEΔD, which expresses inactive DspB, was constructed; this variant effectively serves as a control with the same metabolic burden as EcN SED. This test permits discrimination of the synergistic activity of DspB and S5 (Supplementary Fig. 5). When the expression of S5 pyocin was lowered to the levels observed in the EcN SED system using EcN SEΔD without active DspB as a metabolic control, significantly lower survival of nematodes compared to EcN SE was observed. Because no discernible activity was observed in the presence of DspB alone (EcN dspB), this suggests that DspB may potentiate the activity of S5, resulting in prolonged nematode survival. The *in vivo* potentiation of S5 and the anti-biofilm activity of DspB were more closely examined in a murine infection model.

### Engineered *E. coli* Nissle in a *P. aeruginosa*-infected murine model

**Figure 4:**
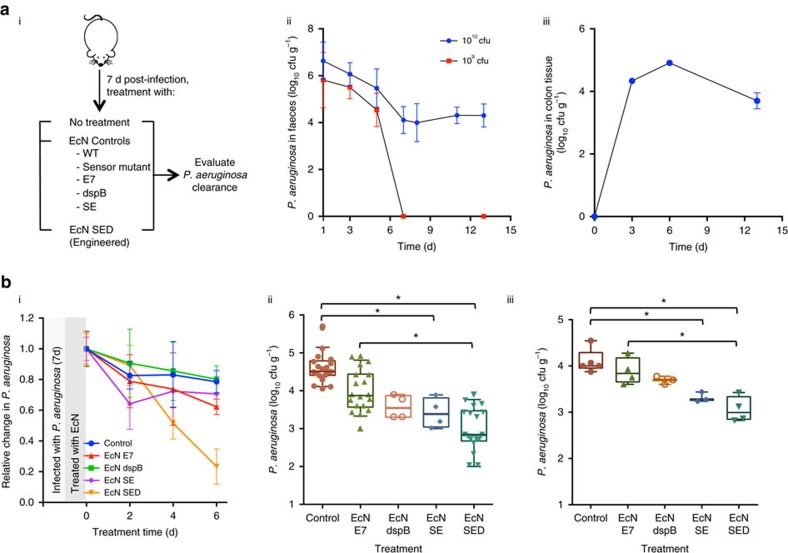
Evaluation of the engineered probiotic strain in a mouse infection model. (**a**) (i) Mice were infected with *P. aeruginosa*, and gut colonization was studied for 7 days. The mice were then given EcN, and *P. aeruginosa* in faeces was quantified and compared with the infection control group. (ii) Colonization of *P. aeruginosa* was assessed by faecal counting of bacteria for two different numbers of cells given by oral gavage. (iii) Colon tissue was homogenized and plated for viability testing to confirm GI infection by *P. aeruginosa* (10^10^ cfu) over time. (**b**) (i) The bacterial count relative to average *P. aeruginosa* infection at day 0 was tracked over 6 days post-treatment with EcN cells. All EcN expressing E7 are shown for comparison with the lysis controls. Other non-lysis EcN controls (EcN wild-type and Sensor mutant) are shown in Supplementary Fig. 6. (ii–iii) Total viable *P. aeruginosa* cell counts from faecal (ii) and (iii) colon samples at day 6 post-treatment. The data from two independent experiments are shown (*n*=8–10). *P<0.01 (Kruskal–Wallis test with Dunn correction). Data are presented in box plots with median as centre line, 10 and 90% confidence limits, and minimum and maximum values.

**Figure 5:**
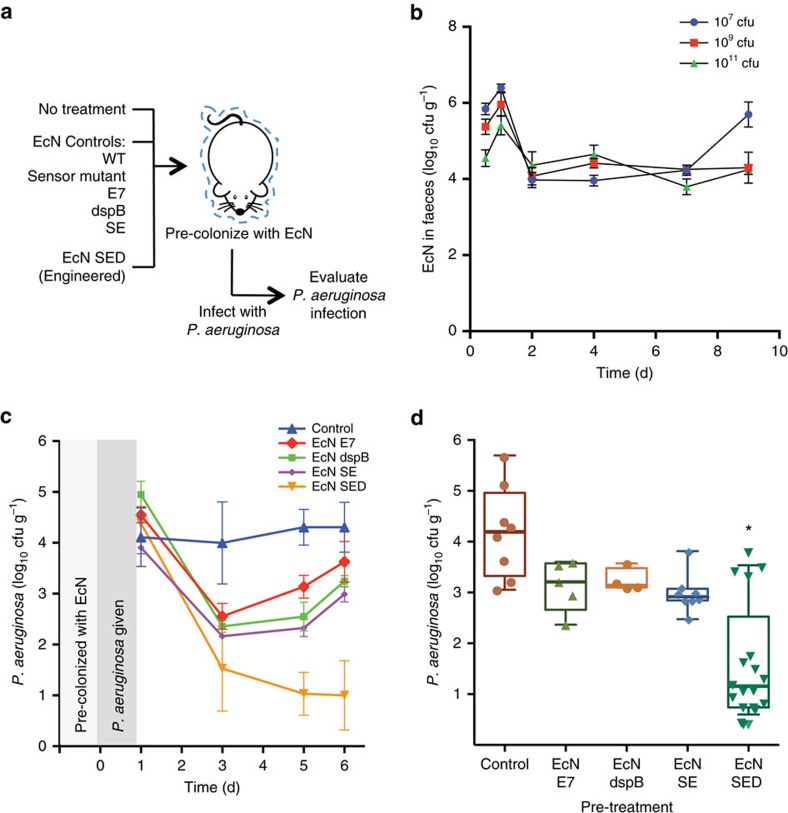
Prophylactic activity of engineered EcN against *P. aeruginosa* infection. (**a**) Scheme for pre-colonization of EcN in the GI tract followed by *P. aeruginosa* infection. (**b**) Colonization of EcN was assessed by faecal counting of bacteria as a function of time in animals that received different numbers of cells by oral gavage. (**c**) The level of *P. aeruginosa* was tracked over 6 days after giving 10^10^ cfu of the pathogen to each group of pretreated mice. (**d**) Total viable *P. aeruginosa* cell counts from faecal samples at day 6 post-infection. The data from two independent experiments are shown (*n*=5–10). * *P*<0.01 (one-way ANOVA with Bonferroni correction for multiple comparisons). Data are presented in box plots with median as centre line, 10 and 90% confidence limits, and minimum and maximum values.

Further evaluation of our engineered EcN was conducted in a *P. aeruginosa*-infected murine model. In this study, a chronic colonization model was used as this allows discrimination of both the cell killing and the biofilm targeting activities that have been engineered into our EcN synthetic biology constructs that, in turn, are evaluated against the pathological processes associated with *P. aeruginosa* infection. We first tested the ability of *P. aeruginosa* to establish chronic colonization in the GI tracts of streptomycin-treated mice over a 13-day period (Fig. 4a). After a single administration of 10^10^ cfu of live cells, the *P. aeruginosa* count stabilized to ∼10^4^ to 10^5^ cfu g^−1^ of faeces and colon tissue after 5 days (Fig. 4a). Likewise, the ability of EcN to colonize the gut was verified (Fig. 5b). When the mice infected with *P. aeruginosa* were fed engineered EcN SED, a steady decline in the *P. aeruginosa* count in the faeces was observed; clearance of the bacterial load reached 77% compared to the initial bacterial load before treatment (Fig. 4b). Only a minor decline over time was observed in mice fed EcN E7 or other controls such as EcN dspB and EcN SE. Interestingly, animals fed wild-type EcN showed a transient decline in bacterial load that initially resembled the pattern of EcN SED, but this reversed on day 6 (Supplementary Fig. 6A). At the end of the experiment, faecal samples and colon tissues were collected for examination (Fig. 4b). A reduction in *P. aeruginosa* levels was observed in the EcN SE and EcN SED treatment groups compared to the infection control group. A minor reduction in *P. aeruginosa* levels was observed in other EcN treatment groups (that is, wild-type, E7 lysis control, dspB and Sensor mutant), although statistical significance was not achieved (Kruskal–Wallis test with Dunn correction, *P*>0.01 for all). Because a minor reduction in bacterial load was observed in the EcN control groups, the activity of EcN SE and EcN SED was compared to that of EcN wild-type (non-lysis control) and E7 (lysis control); only EcN SED achieved a statistically significant reduction in *P. aeruginosa* colonization (Kruskal–Wallis test with Dunn correction).

Next, we evaluated the prophylactic activity of our engineered EcN SED against the onset of infection (Fig. 5). Because our engineered EcN SED is activated only in the presence of *P. aeruginosa* (via the AHL), it should behave in a manner consistent with wild-type Nissle in the absence of the pathogen. This was a fundamental premise in our initial design concept. The colony counts of EcN and EcN SED in pretreatment samples were consistently similar to each other (not shown). Mice were given EcN SED or the respective EcN controls and subsequently subjected to *P. aeruginosa* infection. After 6 days of infection, the extent of *P. aeruginosa* gut colonization was evaluated. Our fully engineered EcN SED provided the greatest protection; in animals treated with EcN SED, lower *P. aeruginosa* cell counts were observed than in the infection alone group (3.2 log_10_ (cfu g^−1^)). A low-level of protection (for example, 1–1.5 log_10_ reduction) was observed in the other pretreatment groups (wild-type, E7 lysis control, dspB, Sensor mutant and SE), although statistical significance was not achieved (one-way ANOVA with Bonferroni multiple comparisons test, significance achieved when *P*<0.008). Administration of EcN SED resulted in 98% inhibition of *P. aeruginosa* infection compared to pretreatment with wild-type EcN. Furthermore, consistent with the results of the *C. elegans* study, the absence of protection against *P. aeruginosa* infection by EcN SE or EcN dspB alone supports the synergistic efficacy of combining anti-microbial and anti-biofilm enzymes to target the two bacterial physiological states (that is, planktonic and biofilm) that are present during the initial phase of *P. aeruginosa* infection.

## Discussion

In this work, we demonstrated both prophylactic and therapeutic efficacy of a functional probiotic microbe *in vivo* against *P. aeruginosa* infection. The probiotic Nissle strain used in this work has been engineered to achieve highly sensitive and enhanced pathogen-activated expression in non-selective conditions. This arrangement enhanced the stability of the high-copy-number plasmid in the absence of the conventional antibiotic resistance genes that are typically required for plasmid retention, while ensuring sufficient expression of our genetic circuit to produce strong killing capacity. Notably, as the auxotrophic strain containing the Δ*alr* Δ*dadX* mutations did not survive in medium devoid of D-alanine without the alr+ plasmid, we suggest that the auxotrophic mutation offers a means of strain biocontainment. When combined with the introduction of the cell-lysis mechanism to the engineered host cells, these features potentially enable a double layer of safety/containment. That is, the engineered cells may promote their own clearance after executing their programmed functionalities in the presence of the pathogen or when the expression construct is lost.

The prophylactic and antimicrobial effects of the engineered Nissle strain against *P. aeruginosa* infection were demonstrated in two animal models. The engineered cells were able to sense the pathogen and accelerate bacterial clearance from the gut in both model systems, and bacterial clearance was visually demonstrated in *C. elegans*. On the basis of both infection models, although Nissle alone may exhibit beneficial effects against *P. aeruginosa*, significant clearance occurred only in the presence of the antimicrobial and anti-biofilm proteins (S5 and DspB) in our engineered strain. We also observed that the initial clearance activity that occurred in *C. elegans* during the first 20 h in the presence of the engineered cells and over a 3- to 4-day period in mice seemed to reflect the eventual survival rate or the extent of clearance of *P. aeruginosa* at later time points. This can be explained by the eventual clearance of the bacteria by our engineered cells upon detection of *P. aeruginosa* due to cell-lysis mediated release of the antimicrobial proteins. This might have prolonged clearance of the pathogen in the EcN SED group beyond 3–4 days post-treatment, but this hypothesis warrants further tests.

Whereas the highest activity against *P. aeruginosa* was demonstrated by EcN SED (S5 and DspB), moderately high activity of EcN SE (S5) was observed in both *in vivo* models. In the *C. elegans* model, we did not find significant differences in survival between the EcN SE and SED treatment groups, suggesting that the overall survival benefit conferred by S5 in combination with DspB could be achieved through a higher expression level of S5 alone. However, it is the combination of S5 and DspB that exhibited strong clearance and prophylactic activity against *P. aeruginosa*. This finding supports the idea that combining anti-microbial and anti-biofilm enzymes produces potentiating activity that enhances pathogen killing efficiency.

We note, however, that the current design is based on the ability of the bacterium to detect the pathogen-associated quorum-signal molecule, and this naturally presents a limitation in the form of a detection threshold. This potential limitation could be offset, however, by recognizing that the engineered *E. coli* can also serve as sentinels or reporters of the pathogen^39^, so that repeated dosing might be signalled and enabled. Furthermore, the current design provides a platform through which the genetic features of individual strains can be reprogrammed to provide other biological functionalities or to combat other pathogens of interest. For this reason, our design exclusively targets pathogens that produce AHL as QS molecules and cells that are susceptible to pyocin S5. Although some *P. aeruginosa* strains are reported to carry immunity genes that confer resistance to this bacteriocin^40^,^41^, the modularity of the genetic circuit used in our design provides a foundation for additional functionalities such as the creation of an S5-immunity inactivation construct or one that causes the bacterium to incorporate complementary antimicrobial peptides.

Last, we suggest that the autonomous nature of our design offers an advantage over conventional antibiotic drug therapies, as the administration of antimicrobial probiotics does not require that infection-associated symptoms be present. These bacteria could potentially be freely administered for their normal probiotic-associated benefits as part of a normal probiotic regimen, while also providing potential prophylactic activity against opportunistic pathogens. In support of this idea, our animal data demonstrate that the engineered strain is more efficient in preventing the onset of an infection than in fighting a pre-established one. That is, protection was provided by a single dose of engineered probiotics administered 7 days before exposure to the pathogen. We anticipate that the duration of protection provided by an engineered probiotic would depend on the ability of the cells to remain in the gut, as well as on many other factors. In the mouse intestine, stable colonization by the Nissle strains for periods of up to 3 weeks was observed^5^,^42^; hence, a single dose of engineered probiotic could potentially provide weeks of protection against the onset of a *P. aeruginosa* infection.

## Methods

### Strains and media

All cloning and characterization experiments were performed in *E. coli* TOP10 (Invitrogen, Carlsbad, CA, USA) unless otherwise stated. Commercial Luria–Bertani (LB) was used as the medium for cloning unless otherwise stated. Kanamycin (30 μg ml^−1^) was added to the culture medium for antibiotic selection where appropriate. *P. aeruginosa* In7, a clinical isolate that is sensitive to S5 pyocin, was used throughout this study^43^. The probiotic *E. coli* Nissle 1917 was obtained from Mutaflor (Canada).

### Generation of deletion mutant and plasmid generation

Chromosomal sequences comprising the targeted genes *alr* and *dadX* were replaced using the Wanner Lambda Red Gene Disruption kit from the Coli Genetic Stock Center^33^. The primers used for construction of the deletion mutants are listed in Supplementary Table 2. Mutant candidates were tested for the loss of the target gene by PCR. In addition, mutants were sequenced for genotype confirmation and tested for growth phenotype. The medium was supplemented with D-alanine (50 μg ml^−1^) for the growth of the Δ*alr* Δ*dadX* strain (not carrying the alr+ plasmid). The genetic constructs developed in this study were assembled using standard synthetic biology protocols^44^ and introduced into the vector pBbE8K (ref. 34), which was modified to include a ColE1 replication origin, *alr* for auxotrophic complementation, and FRT-flanked *KanR*, (herein referred to as pEaaK). A GFP expression cassette was cloned into pEaaK, and both wild-type and EcN deletion strains were transformed with the plasmid. The transformed cells were grown in tryptone broth in the absence of antibiotic selection and diluted 10,000-fold into fresh medium every 24 h over a 30-day period. GFP expression and *KanR* phenotype were measured at 10-day intervals as indicators of plasmid retention.

### Characterization of GFP expression constructs with 3OC_12_-HSL

Single colonies of *E. coli* containing the constructs were inoculated into LB. After overnight growth, the cultures were diluted into fresh LB to a low OD and allowed to incubate further to OD_600_ of 0.5. The cultures were then transferred to clear polystyrene 96-well plates in triplicate 200-μl aliquots for induction with homoserine lactone, 3OC_12_-HSL (AHL; Sigma-Aldrich) at varying molar concentrations in 10-fold increments from 10^−9^ M to 10^−5^ M for 6 h. The plate was incubated at 37 °C with moderate shaking in a microplate reader (Biotek Instruments, USA) and assayed for green fluorescence (excitation; 485 nm and emission; 528 nm). Time-series fluorescence and OD_600_ data were obtained over a period of 6 h. The relative GFP production rate was defined as the ratio of background subtracted green fluorescence to OD_600_ value. The results obtained from constructs expressed in the Nissle wild-type and the double mutant (Nissle Δ*alr* Δ*dadX*—EcN) strains were plotted in MATLAB.

### Enzyme assay of DspB protein expressed in Nissle

A synthetic substrate, 4-nitrophenyl-N-acetyl-β-D-glucosaminide, was used to test the enzymatic activity of DspB. A supernatant was prepared by inducing the expression of DspB and E7 in Nissle for 4 h with 100 nM AHL at an OD_600_ of 0.6. Under these conditions, the cells lyse due to E7 expression, releasing DspB into the supernatant. Enzyme reactions were carried out in a 100-μl volume containing 4 mM substrate in PBS buffer and 10 μg ml^−1^ cellular supernatant; the change in absorption of the supernatant at 405 nm due to the release of p-nitrophenolate was immediately measured. The protein concentration of the supernatant was determined using a standard Bradford assay.

### Assay for biofilm detachment of *P. aeruginosa* biofilm treated with engineered EcN

Bacterial biofilms were formed by immersing the pegs of a modified polystyrene microtiter lid (catalogue no. 4,45,497; Nunc TSP system)^45^ into a 96-well microtiter plate in which each well had been filled with 150 μl of medium containing a single-cell suspension of *P. aeruginosa* at an OD_600_ of 0.05. The plates were incubated at 37 °C for 24 h. The wells were rinsed with PBS, DspB supernatant or engineered EcN (E7 and SED) was added to each well, and the plates were incubated for an additional 4 h. The wells were washed thrice with PBS; the bacteria that remained attached to the surface were stained with crystal violet (0.1% w/v), rewashed under running tap water and dried. The amount of bio-film mass was quantified by destaining the bio-films for 20 min with 95% ethanol and then measuring the absorbance of the crystal violet solution at 595 nm. To assess the number of viable cells within the biofilm, previously published methods were adopted^46^ in which the pegs were transferred onto a biofilm recovery plate (a flat-bottom microtiter plate containing antibiotic-free medium). The recovery plate was centrifuged at 805*g* for 20 min, and the collected cultures were processed for counting of colony-forming units.

### Cell viability testing of co-cultured engineered EcN and *P. aeruginosa*

Overnight cultures of *P. aeruginosa* (bearing a plasmid that conferred constitutive expression of GFP; pMRP9-1)^47^, and engineered Nissle were diluted and harvested at the exponential phase. EcN containing the constructs (E7 or SED) were added to *P. aeruginosa* cultures (final OD_600_ 0.2) in appropriate ratios. As *P. aeruginosa* In7 constitutively expresses GFP from the plasmid, GFP fluorescence was detected using a microplate reader over time to measure the growth retardation. EcN containing the E7 construct (EcN-E7) served as the negative control for EcN SED. For cell viability assays, aliquots of In7 in the mixed culture were quantified by colony-forming unit (cfu) counting on carbenicillin-selective agar plates at regular intervals ranging from 4 to 12 h. Percentage survival of *P. aeruginosa* was tabulated relative to EcN-E7.

### Microscopy of biofilms

Mixed bacterial cultures of GFP-expressing *P. aeruginosa* and engineered EcN (SED and its control variants) were cultured in 8-well chamber slides (SPL Lifesciences) in antibiotic-free medium. The biofilm that developed on the glass slides after 18–20 h of growth was rinsed in PBS, dried and visualized using an LSM 510 confocal laser scanning microscope (Zeiss, Jena, Germany). The collected Z-stack biofilm images were reconstructed using ImageJ software. Likewise, biofilm detachment images were prepared after treating the mature biofilms (obtained at 24–48 h of bacterial growth) with engineered EcN for 6 h. The same procedures were repeated with other control variants of EcN as negative controls.

### *Caenorhabditis elegans* killing assay

The *C. elegans* strain AU37 used in this study was obtained from the Caenorhabditis Genetic Center (http://www.cbs.umn.edu/cgc). AU37 is a pathogen-sensitive strain that exhibits temperature-sensitive sterilization. All nematode-related maintenance protocols were performed according to Powell and Ausubel (ref. 48). The worms were allowed to grow at 16 °C for maintenance or at room temperature for sterility or for assays. The pathogenicity killing assay was performed with modifications. Adult nematodes were infected on slow-killing plates (SKPs) containing a *P. aeruginosa* (with pMRP9-1) bacterial lawn for 24 h. They were then washed off the plates with M9 buffer, collected by centrifugation, and washed two additional times in M9 buffer containing 200 μg ml^−1^ kanamycin followed by resuspension in M9 buffer. The nematodes were then transferred to SKP plates containing an *E. coli* bacterial lawn for treatment. The nematodes were observed under fluorescence microscope, and the percentage (%) survival of *C. elegans* was tabulated. At least three replicates of ∼100 worms were carried out. The half-maximal lethal time (LT_50_) for each treatment was modelled in MATLAB and calculated as described in ref. 38. For imaging and GFP fluorescence measurement, the nematodes were first anesthetized with 1.5 mM sodium azide (NaN_3_), then placed on agar and imaged for GFP fluorescence. The fluorescence intensity of 10–20 worms from each treatment group was quantified using ImageJ. Representative individuals were photographed to illustrate the localization of the bacteria.

### Animal model

Studies were performed using 6- to 8-week-old female ICR mice in accordance with international ethical guidelines. All procedures were conducted under IACUC guidelines and in conformity with protocols approved by the SingHealth Institutional Animal Care and Use Committee (2013/SHS/810). Outbred female ICR mice (6–8 weeks old) were given drinking water containing streptomycin sulfate (2 mg ml^−1^) and penicillin (1,500 U penicillin G ml^−1^) for 4 days to eliminate residing facultative bacteria. After 1 day of rest, food was withdrawn overnight, and a single dose of *P. aeruginosa* (10^10^ cfu) in 20% sucrose solution was administered to the mice via oral gavage. Stool samples were collected at the indicated time points and homogenized in 1 ml of 1% protease peptone, serially diluted in this medium and plated on cetrimide agar containing carbenicillin to measure levels of GI colonization. Selection was made against *P. aeruginosa* harbouring pMRP9.1 plasmid. The mice were randomly subdivided into various EcN treatment groups (wild-type, E7 lysis control and SED-engineered); treatment was given via oral gavage (10^10^ cfu). Viability assays of the animals' faeces were subsequently performed to evaluate the kinetics of clearance of viable *P. aeruginosa* from the GI tract. Enumeration was performed in a blinded fashion in which the treatment information was unknown.

For the prophylactic model, the mice were fed EcN containing pEaaK plasmid or its derivatives (10^10^ cfu) in 20% sucrose solution and GI colonization of EcN before *P. aeruginosa* infection was verified by viability assays of the faeces, using kanamycin for selection. On day 6, the mice were challenged with *P. aeruginosa* (10^10^ cfu), and their subsequent GI colonization was monitored by counting *P. aeruginosa* in faeces. Each colonization experiment was performed at least twice with 5–10 mice with essentially identical results. Pooled data from at least two independent experiments are presented in the figures.

### Statistics and reproducibility of results

All microtiter plate assays (GFP reporter and biofilm assays) were performed in triplicate wells; the triplicate samples exhibited <10% variation in absorbance and fluorescence values. All assays were performed 2–3 times with similarly significant differences in the quantified values. Results are presented as mean±s.e.m., unless stated otherwise. Student's *t*-test and one-way ANOVA were used for statistical analysis involving two groups or multiple experimental group comparisons, respectively. When the sample distributions were not normal, a nonparametric Kruskal–Wallis test was performed. Bonferroni's correction was performed for multiple comparisons with the significance level α value set to 0.05.

The Kaplan–Meier method was applied using GraphPad Prism software to obtain the survival fractions of infected *C. elegans* treated with variants of EcN. The statistical differences between the survival rates of the EcN-treated groups and the infection control group were determined using the Mantel–Haenszel log-rank test.

### Data availability

The authors declare that all relevant data supporting the findings of this study are available within the article and its Supplementary Information Files or from the corresponding author on request.

## Additional information

**How to cite this article:** Hwang, I. Y. *et al*. Engineered probiotic *Escherichia coli* can eliminate and prevent *Pseudomonas aeruginosa* gut infection in animal models. *Nat. Commun.* **8,** 15028 doi: 10.1038/ncomms15028 (2017).

**Publisher's note:** Springer Nature remains neutral with regard to jurisdictional claims in published maps and institutional affiliations.

## Supplementary Material

Supplementary Information Supplementary Tables, Supplementary Figures and Supplementary Reference

## References

[b1] Renwick M. J., Brogan D. M. & Mossialos E. A systematic review and critical assessment of incentive strategies for discovery and development of novel antibiotics. J. Antibiotics 69, 73–88 (2015). 26464014 10.1038/ja.2015.98 PMC4775540

[b2] Ukena S. N. . Probiotic *Escherichia coli* Nissle 1917 inhibits leaky gut by enhancing mucosal integrity. PLoS ONE 2, e1308–e1308 (2007). 18074031 10.1371/journal.pone.0001308 PMC2110898

[b3] Schultz M. Clinical use of *E. coli* Nissle 1917 in inflammatory bowel disease. Inflamm. Bowel Dis. 14, 1012–1018 (2008). 18240278 10.1002/ibd.20377

[b4] Westendorf A. M. . Intestinal immunity of *Escherichia coli* NISSLE 1917: a safe carrier for therapeutic molecules. FEMS Immunol. Med. Microbiol. 43, 373–384 (2005). 15708311 10.1016/j.femsim.2004.10.023

[b5] Leatham M. P. . Precolonized human commensal *Escherichia coli* strains serve as a barrier to *E. coli* O157:H7 growth in the streptomycin-treated mouse intestine. Infect. Immunity 77, 2876–2886 (2009). 19364832 10.1128/IAI.00059-09 PMC2708557

[b6] Hancock V., Dahl M. & Klemm P. Probiotic *Escherichia coli* strain Nissle 1917 outcompetes intestinal pathogens during biofilm formation. J. Med. Microbiol. 59, 392–399 (2010). 20110388 10.1099/jmm.0.008672-0

[b7] Goh Y.-L., He H. & March J. C. Engineering commensal bacteria for prophylaxis against infection. Curr. Opin. Biotechnol. 23, 924–930 (2012). 22459613 10.1016/j.copbio.2012.03.004 PMC3389292

[b8] Duval M. . Enhanced neutralization of HIV by antibodies displayed on the S-layer of *Caulobacter crescentus*. Antimicrob. Agents Chemother. 55, 5547–5552 (2011). 21896905 10.1128/AAC.00509-11 PMC3232825

[b9] Farr C. . Development of an HIV-1 microbicide based on: blocking infection by high-density display of virus entry inhibitors. PLoS ONE 8, e65965 (2013). 23840383 10.1371/journal.pone.0065965 PMC3686833

[b10] Volzing K., Borrero J., Sadowsky M. J. & Kaznessis Y. N. Antimicrobial peptides targeting Gram-negative pathogens, produced and delivered by lactic acid bacteria. ACS Synth. Biol. 2, 643–650 (2013). 23808914 10.1021/sb4000367 PMC4222081

[b11] Gupta S., Bram E. E. & Weiss R. Genetically programmable pathogen sense and destroy. ACS Synth. Biol. 2, 715–723 (2013). 23763381 10.1021/sb4000417

[b12] Lu T. K. & Collins J. J. Engineered bacteriophage targeting gene networks as adjuvants for antibiotic therapy. Proc. Natl Acad. Sci. 106, 4629–4634 (2009). 19255432 10.1073/pnas.0800442106 PMC2649960

[b13] Duan F. F., Liu J. H. & March J. C. Engineered commensal bacteria reprogram intestinal cells into glucose-responsive insulin-secreting cells for the treatment of diabetes. Diabetes 64, 1794–1803 db140635-db140635 (2015). 25626737 10.2337/db14-0635 PMC4407861

[b14] Duan F. & March J. C. Engineered bacterial communication prevents *Vibrio cholerae* virulence in an infant mouse model. Proc. Natl Acad. Sci. USA 107, 11260–11264 (2010). 20534565 10.1073/pnas.1001294107 PMC2895089

[b15] Din M. O. . Synchronized cycles of bacterial lysis for *in vivo* delivery. Nature 536, 81–85 (2016). 27437587 10.1038/nature18930 PMC5048415

[b16] St Jean A. T., Swofford C. A., Panteli J. T., Brentzel Z. J. & Forbes N. S. Bacterial delivery of *Staphylococcus aureus* alpha-hemolysin causes regression and necrosis in murine tumors. Mol. Ther. 22, 1266–1274 (2014). 24590046 10.1038/mt.2014.36 PMC4089002

[b17] Smith E. E. . Genetic adaptation by *Pseudomonas aeruginosa* to the airways of cystic fibrosis patients. Proc. Natl Acad. Sci. 103, 8487–8492 (2006). 16687478 10.1073/pnas.0602138103 PMC1482519

[b18] Markou P. & Apidianakis Y. Pathogenesis of intestinal *Pseudomonas aeruginosa* infection in patients with cancer. Front. Cell. Infect. Microbiol. 3, 115 (2013). 10.3389/fcimb.2013.00115 PMC3882663 24432250

[b19] Rolston K. V. & Bodey G. P. *Pseudomonas aeruginosa* infection in cancer patients. Cancer Investig. 10, 43–59 (1992). 1735012 10.3109/07357909209032787

[b20] Alverdy J. . Gut-derived sepsis occurs when the right pathogen with the right virulence genes meets the right host: evidence for *in vivo* virulence expression in *Pseudomonas aeruginosa*. Ann. Surg. 232, 480–489 (2000). 10998646 10.1097/00000658-200010000-00003 PMC1421180

[b21] Watanabe R. . Efficacy of bacteriophage therapy against gut-derived sepsis caused by *Pseudomonas aeruginosa* in mice. Antimicrob. Agents Chemother. 51, 446–452 (2007). 17116686 10.1128/AAC.00635-06 PMC1797723

[b22] Marshall J. C., Christou N. V. & Meakins J. L. The gastrointestinal tract. The ‘undrained abscess' of multiple organ failure. Ann. Surg. 218, 111–119 (1993). 8342990 10.1097/00000658-199308000-00001 PMC1242919

[b23] de Jonge E. . Effects of selective decontamination of digestive tract on mortality and acquisition of resistant bacteria in intensive care: a randomised controlled trial. Lancet 362, 1011–1016 (2003). 14522530 10.1016/S0140-6736(03)14409-1

[b24] Zaborina O. . Identification of multi-drug resistant *Pseudomonas aeruginosa* clinical isolates that are highly disruptive to the intestinal epithelial barrier. Ann. Clin. Microbiol. Antimicrob. 5, 14–14 (2006). 16762075 10.1186/1476-0711-5-14 PMC1513249

[b25] Okuda J. . Translocation of *Pseudomonas aeruginosa* from the intestinal tract is mediated by the binding of ExoS to an Na,K-ATPase regulator, FXYD3. Infect. Immunity 78, 4511–4522 (2010). 20805335 10.1128/IAI.00428-10 PMC2976341

[b26] Koh A. Y., Priebe G. P. & Pier G. B. Virulence of *Pseudomonas aeruginosa* in a murine model of gastrointestinal colonization and dissemination in neutropenia. Infect. Immunity 73, 2262–2272 (2005). 15784570 10.1128/IAI.73.4.2262-2272.2005 PMC1087461

[b27] Chuang C.-H. . Shanghai fever: a distinct *Pseudomonas aeruginosa* enteric disease. Gut 63, 736–743 (2014). 23943780 10.1136/gutjnl-2013-304786 PMC3995289

[b28] Rowe M. I., Reblock K. K., Kurkchubasche A. G. & Healey P. J. Necrotizing enterocolitis in the extremely low birth weight infant. J. Pediatr. Surg. 29, 987–990 discussion 990-981 (1994). 7965535 10.1016/0022-3468(94)90264-x

[b29] Saeidi N. . Engineering microbes to sense and eradicate *Pseudomonas aeruginosa*, a human pathogen. Mol. Syst. Biol. 7, 521–521 (2011). 21847113 10.1038/msb.2011.55 PMC3202794

[b30] Itoh Y. . Depolymerization of β-1,6-N-Acetyl-D-Glucosamine disrupts the integrity of diverse bacterial biofilms. J. Bacteriol. 187, 382–387 (2005). 15601723 10.1128/JB.187.1.382-387.2005 PMC538831

[b31] Walsh C. T. Enzymes in the D-alanine branch of bacterial cell wall peptidoglycan assembly. J. Biol. Chem. 264, 2393–2396 (1989). 2644260

[b32] Wild J., Hennig J., Lobocka M., Walczak W. & Klopotowski T. Identification of the dadX gene coding for the predominant isozyme of alanine racemase in *Escherichia coli* K12. Mol. Gen. Genet. 198, 315–322 (1985). 3920477 10.1007/BF00383013

[b33] Datsenko K. A. & Wanner B. L. One-step inactivation of chromosomal genes in *Escherichia coli* K-12 using PCR products. Proc. Natl Acad. Sci. USA 97, 6640–6645 (2000). 10829079 10.1073/pnas.120163297 PMC18686

[b34] Lee T. . BglBrick vectors and datasheets: a synthetic biology platform for gene expression. J. Biol. Eng. 5, 12–12 (2011). 21933410 10.1186/1754-1611-5-12 PMC3189095

[b35] Scott M., Gunderson C. W., Mateescu E. M., Zhang Z. & Hwa T. Interdependence of cell growth and gene expression: origins and consequences. Science 330, 1099–1102 (2010). 21097934 10.1126/science.1192588

[b36] Wessely F. . Optimal regulatory strategies for metabolic pathways in *Escherichia coli* depending on protein costs. Mol. Syst. Biol. 7, 515 (2011). 21772263 10.1038/msb.2011.46 PMC3159982

[b37] Bentley W. E., Mirjalili N., Andersen D. C., Davis R. H. & Kompala D. S. Plasmid-encoded protein: the principal factor in the ‘metabolic burden' associated with recombinant bacteria. Biotechnol. Bioeng. 35, 668–681 (1990). 18592563 10.1002/bit.260350704

[b38] Tan M. W., Mahajan-Miklos S. & Ausubel F. M. Killing of *Caenorhabditis elegans* by *Pseudomonas aeruginosa* used to model mammalian bacterial pathogenesis. Proc. Natl Acad. Sci. USA 96, 715–720 (1999). 9892699 10.1073/pnas.96.2.715 PMC15202

[b39] Terrell J. L. . Nano-guided cell networks as conveyors of molecular communication. Nat. Commun. 6, 8500 (2015). 26455828 10.1038/ncomms9500 PMC4633717

[b40] Parret A., De Mot R. & Pavel D. Novel bacteriocins with predicted tRNase and pore-forming activities in *Pseudomonas aeruginosa* PAO1. Mol. Microbiol. 35, 1995–1998 (2000). 10.1046/j.1365-2958.2000.01716.x 10652108

[b41] Rasouliha B. H., Ling H., Ho C. L. & Chang M. W. A predicted immunity protein confers resistance to Pyocin S5 in a sensitive strain of *Pseudomonas aeruginosa*. ChemBioChem 14, 2444–2446 (2013). 24222552 10.1002/cbic.201300410

[b42] Maltby R., Leatham-Jensen M. P., Gibson T., Cohen P. S. & Conway T. Nutritional basis for colonization resistance by human commensal *Escherichia coli* Strains HS and Nissle 1917 against *E. coli* O157:H7 in the mouse intestine. PLoS ONE 8, e53957–e53957 (2013). 23349773 10.1371/journal.pone.0053957 PMC3547972

[b43] Ling H., Saeidi N., Rasouliha B. H. & Chang M. W. A predicted S-type pyocin shows a bactericidal activity against clinical *Pseudomonas aeruginosa* isolates through membrane damage. FEBS Lett. 584, 3354–3358 (2010). 20580355 10.1016/j.febslet.2010.06.021

[b44] Canton B., Labno A. & Endy D. Refinement and standardization of synthetic biological parts and devices. Nat. Biotechnol. 26, 787–793 (2008). 18612302 10.1038/nbt1413

[b45] Ceri H., Olson M. E. & Stremick C. The calgary biofilm device: new technology for rapid determination of antibiotic susceptibilities of bacterial biofilms. J. Clin. Microbiol. 37, 1771–1776 (1999). 10325322 10.1128/jcm.37.6.1771-1776.1999 PMC84946

[b46] Moskowitz S. M., Foster J. M., Emerson J., Burns J. L. & Icrobiol J. C. L. I. N. M. Clinically feasible biofilm susceptibility assay for isolates of *Pseudomonas aeruginosa* from patients with cystic fibrosis. J. Clin. Microbiol. 42, 1915–1922 (2004). 15131149 10.1128/JCM.42.5.1915-1922.2004 PMC404629

[b47] Davies D. G. . The involvement of cell-to-cell signals in the development of a bacterial biofilm. Science 280, 295–298 (1998). 9535661 10.1126/science.280.5361.295

[b48] Powell J. R. & Ausubel F. M. Models of *Caenorhabditis elegans* infection by bacterial and fungal pathogens. Methods Mol. Biol. 415, 403–427 (2008). 18370168 10.1007/978-1-59745-570-1_24

