# Supplementary material for: Engineered probiotic Escherichia coli can eliminate and prevent Pseudomonas aeruginosa gut infection in animal models
Source: Nat Commun. 2017 Apr 11;8:15028. doi: 10.1038/ncomms15028 (PMC5394271; doi:10.1038/ncomms15028)
Supplement: Supplementary Information — Supplementary Tables, Supplementary Figures and Supplementary Reference [file ncomms15028-s1.pdf]

Supplementary Table 1. List of all plasmids used in this study

Name	Plasmid Name	Description of gene expressed	Origin of replication; Selection marker
QS reporter	V1	Constitutive LasR (pTet)<br>pLux controlled GFP	pMB1; Ampicillin
	V5	Constitutive LasR (J23108)<br>pLas controlled GFP	ColE1; alr<br>(opt: Kanamycin)
Killing circuit and Controls	E7	Constitutive LasR (J23108)<br>pLas controlled E7	ColE1; alr<br>(opt: Kanamycin)
	dspB	Constitutive LasR (J23108)<br>pLas controlled DspB, E7	
	Sensor	Constitutive LasR (J23108)<br>pLas controlled mRFP1	
	Sensor mutant	pLas controlled S5, E7, DspB<br>without LasR	
	SE	Constitutive LasR (J23108)<br>pLas controlled S5, E7	
	SED	Constitutive LasR (J23108)<br>pLas controlled S5, E7, DspB	
	SEΔD	Constitutive LasR (J23108)<br>pLas controlled S5, E7, $\Delta DspB_{41\text{-}249}$	

Supplementary Table 2. List of primers used for gene deletion

Primer	Sequence
5’ *alr* homology	Fwd 5’ GTGACGTTGTTATCACCAGGTTTAAACGAT 3’
	Rev 5’ CAGGTTCTGCCCACCAGTGCAAAACCTCGC 3’
3’ *alr* homology	Fwd 5’ CAGGGAGGGACAATGTCTTATTCAGAAATC 3’
	Rev 5’ TCAGGTTTAAACCGCGCGTCATATACAGTG 3’
5’ *dadX* homology	Fwd 5’ ATCCGCTGAAAGGCTACTCGCTGACTATTC 3’
	Rev 5’ ATCTCGTTTCCTTAGCTGTGTGCGCCATGT 3’
3’ *dadX* homology	Fwd 5’ GACGGTGTAACTTGTTATCGCTGGATGCGA 3’
	Rev 5’ CTGCGTGATTTGCATATGCCAAAAGAGACG 3’

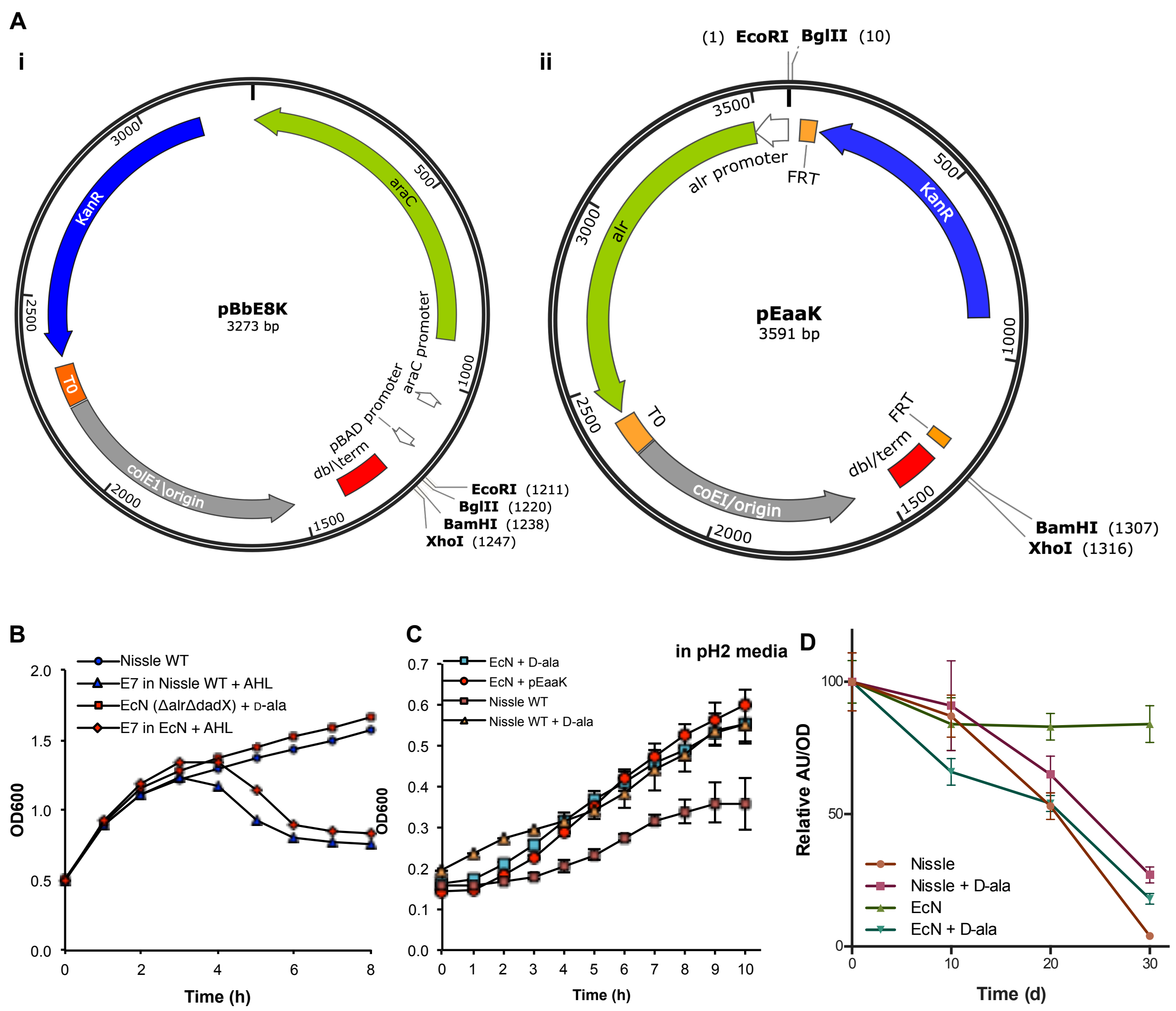

Supplementary Figure 1: **Auxotrophic, non-antibiotic marker Vector-host system**

A. Plasmid maps of (i) a standard BglBrick vector[1] in which the complementary auxotrophic vector was derived from, and (ii) alr+ plasmid pEaaK containing kanamycin resistance gene (*KanR*). pEaaK was used as a backbone to contain the genetic construct for 'sense and kill'. **B.** EcN (Nissle Δalr ΔdadB) QS regulated E7 expression was tested for cell lysis upon induction. **C.** EcN and wild-type Nissle were cultured in low pH media (equivalent to gastric condition) and its growth rate was observed over time. **D.** EcN with pEaaK+GFP (EcN) and wild-type Nissle with pBbE8K+GFP (Nissle) were cultured with or without exogenous D-alanine in non-selective minimal media and GFP expression (measured in arbitrary unit (AU)) was observed over time as an indicator of plasmid

stability. All microtiter plate assays were performed in triplicate wells, and mean and error bar (s.e.m.) from three experiment are shown.

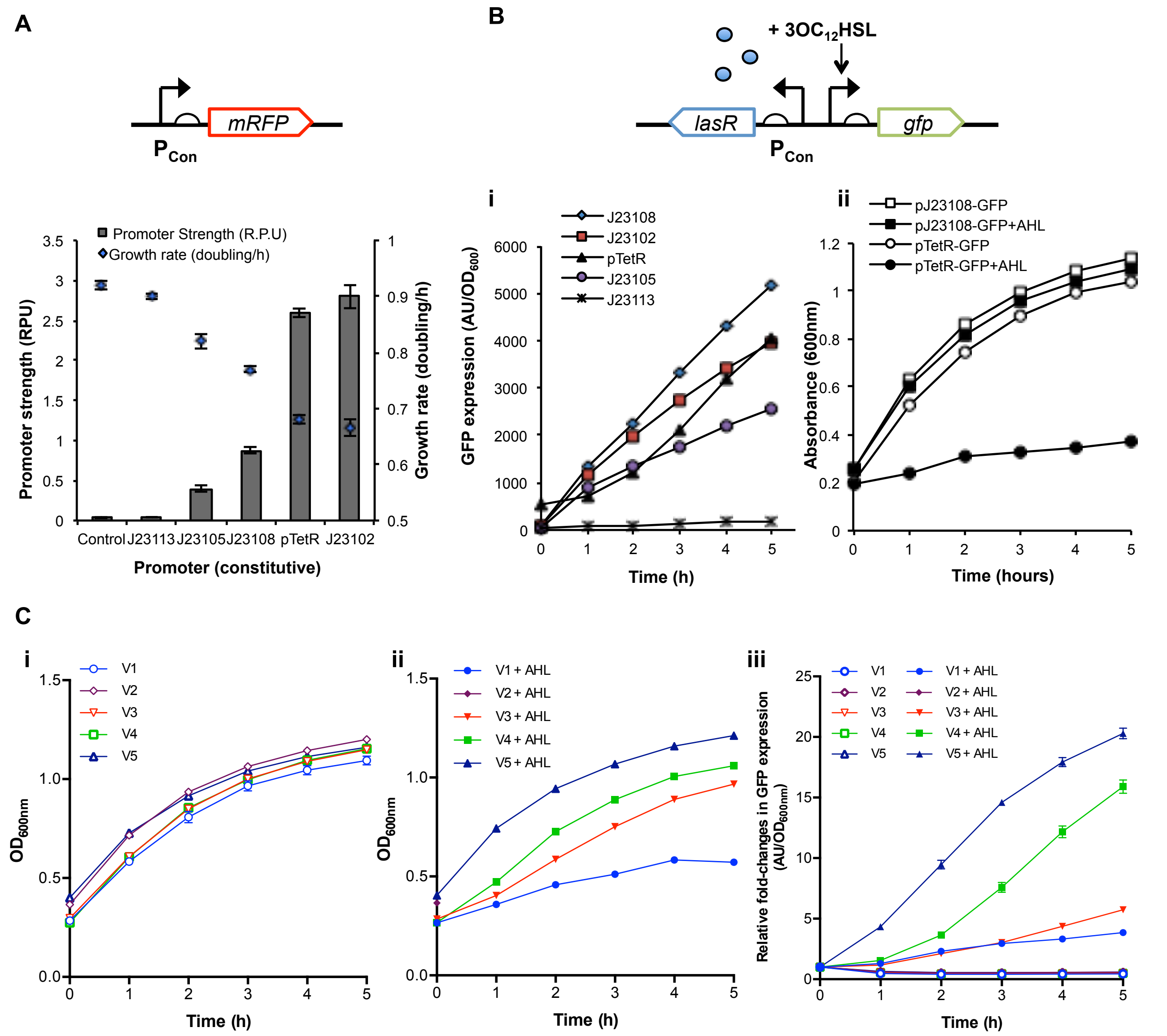

Supplementary Figure 2: Optimization of sensing device expression

A. To modulate the expression of lasR without creating a growth deficit, the constitutive promoter of varying strength (relative promoter unit; fluorescence was normalized to RFP expression driven by promoter of a housekeeping gene, *rrnB* gene) was compared with cellular growth rate (doubling time). J23105 and J23108 showed moderate expression without sustaining significant growth deficit. **B.** When the constitutive promoters expressing lasR was coupled to lasR-activated GFP expression (Quorum Sensing (QS) reporter), J23108 promoter resulted in highest GFP expression rate. When the cellular growth was observed, J23108 promoter driven QS reporter resulted in growth

that appears to be unaffected in activated state induced by quorum sensing molecule (N-Acyl homoserine lactone; AHL). **C.** Systematic optimization of expression vector (V1~5 from Figure 1) shows improved growth upon AHL induction (10^{-6}M). Cell growth under (i) un-induced and (ii) induced condition was monitored over time. Relative fold increase in GFP expression normalized to cell density (OD_{600nm}) upon AHL induction shows that V5 shows most robust expression. All microtiter plate assays were performed in triplicate wells, and mean and error bar (s.e.m.) from three experiment are shown.

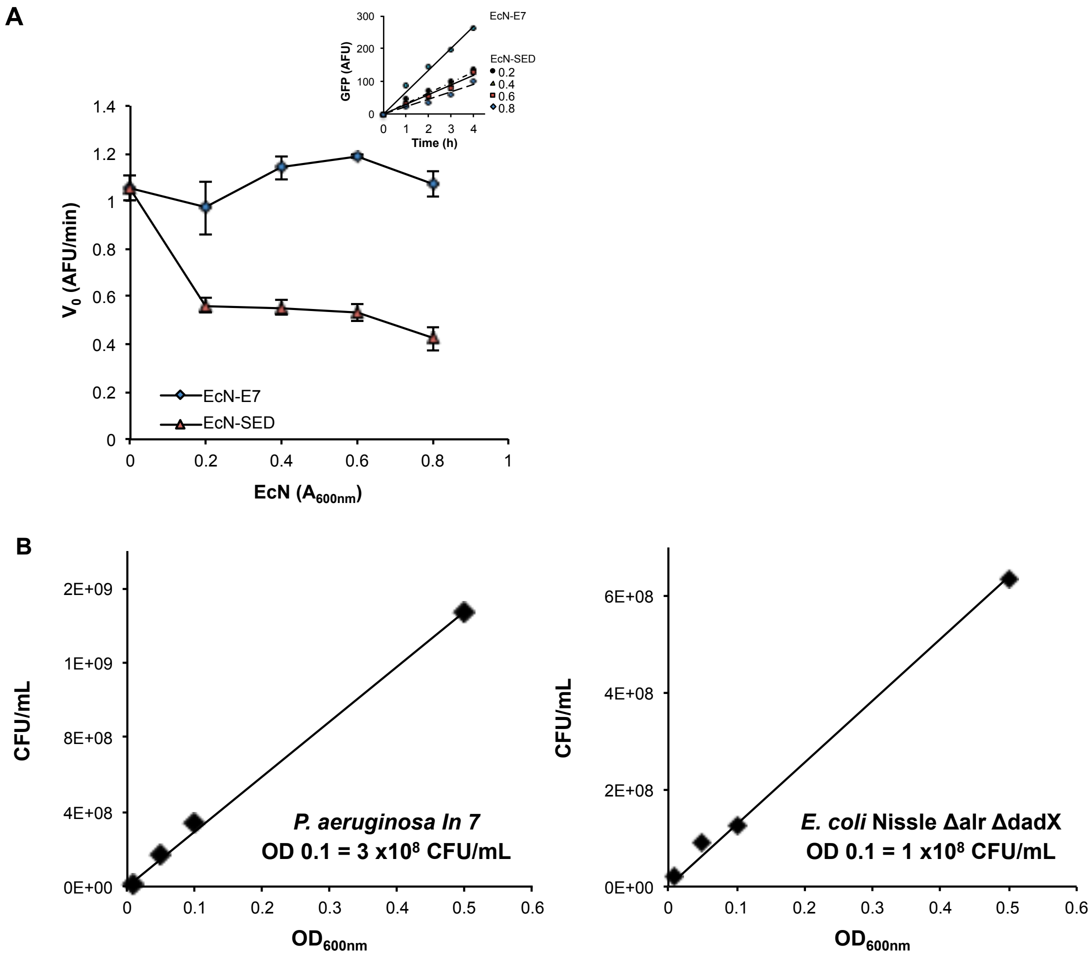

Supplementary Figure 3: ***In vitro*** **evaluation of engineered** ***E. coli*** **Nissle against** ***P. aeruginosa*****.** **A.** The effect on growth rate of *P. aeruginosa* (reflected by GFP expression) was measured over time when cultured together with engineered EcN containing SED or E7 (lysis control). *Inset graph: GFP value was normalized to the start of the exponential phase. All microtiter plate assays were performed in triplicate wells, and mean and error bar (s.e.m.) from three experiment are shown. **B.** Correlation between the optical density and colony forming units (cfu) of *P. aeruginosa* In7 and *E. coli* Nissle Δalr ΔdadX.

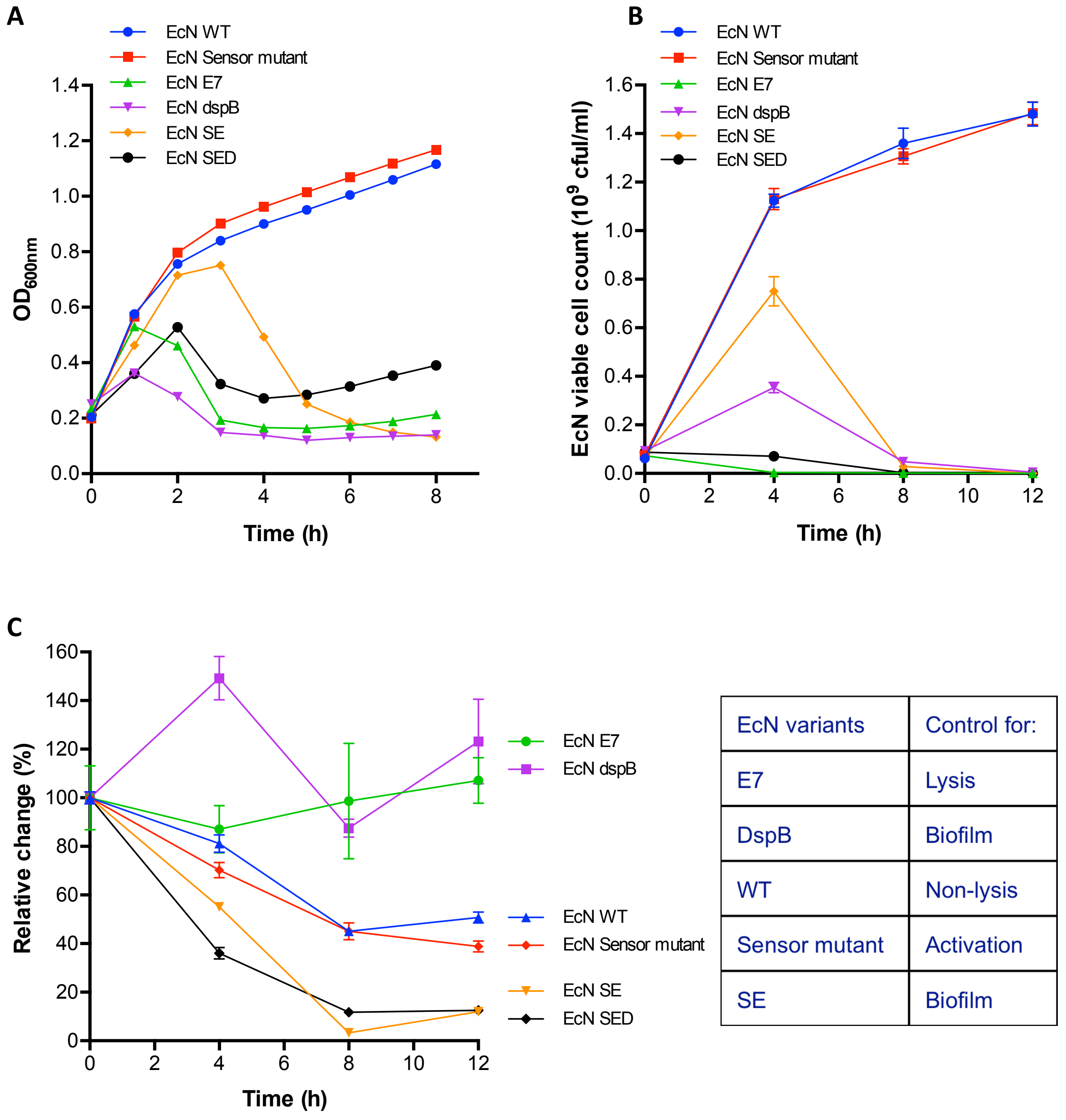

EcN variants	Control for:
E7	Lysis
DspB	Biofilm
WT	Non-lysis
Sensor mutant	Activation
SE	Biofilm

Supplementary Figure 4: Co-culturing of *P. aeruginosa* and EcN

A. Growth profile of EcN control variants upon AHL induction. Cells were induced with 0.1µM AHL and subsequent growth was observed over time. All microtiter plate assays were performed in triplicate wells, and mean and error bar (s.e.m.) from three experiment are shown. **B**. Viability of EcN at the given time point of co-culture with *P. aeruginosa* (PA). EcN dspB (DspB & E7), EcN SE (S5 & E7) and EcN SED (S5, E7 and DspB). **C**. Viability of PA when co-cultured with control variants of engineered EcN over time. The control variants include; EcN WT, EcN Sensor mutant

(contains genetic construct of SED, under mutated PA sensor, lasR) and EcN E7 (lysis control). A table summarizes each EcN variant and its purpose as control for SED group. It is observed that while EcN expressing E7 results in cell lysis, wild-type and sensor mutant cells showed constant growth over time. This growth may allow EcN cells to outcompete and cause reduced *P. aeruginosa* cell growth in co-culture condition. The mean and error bar (s.e.m.) from three experiment is shown for all viability assays.

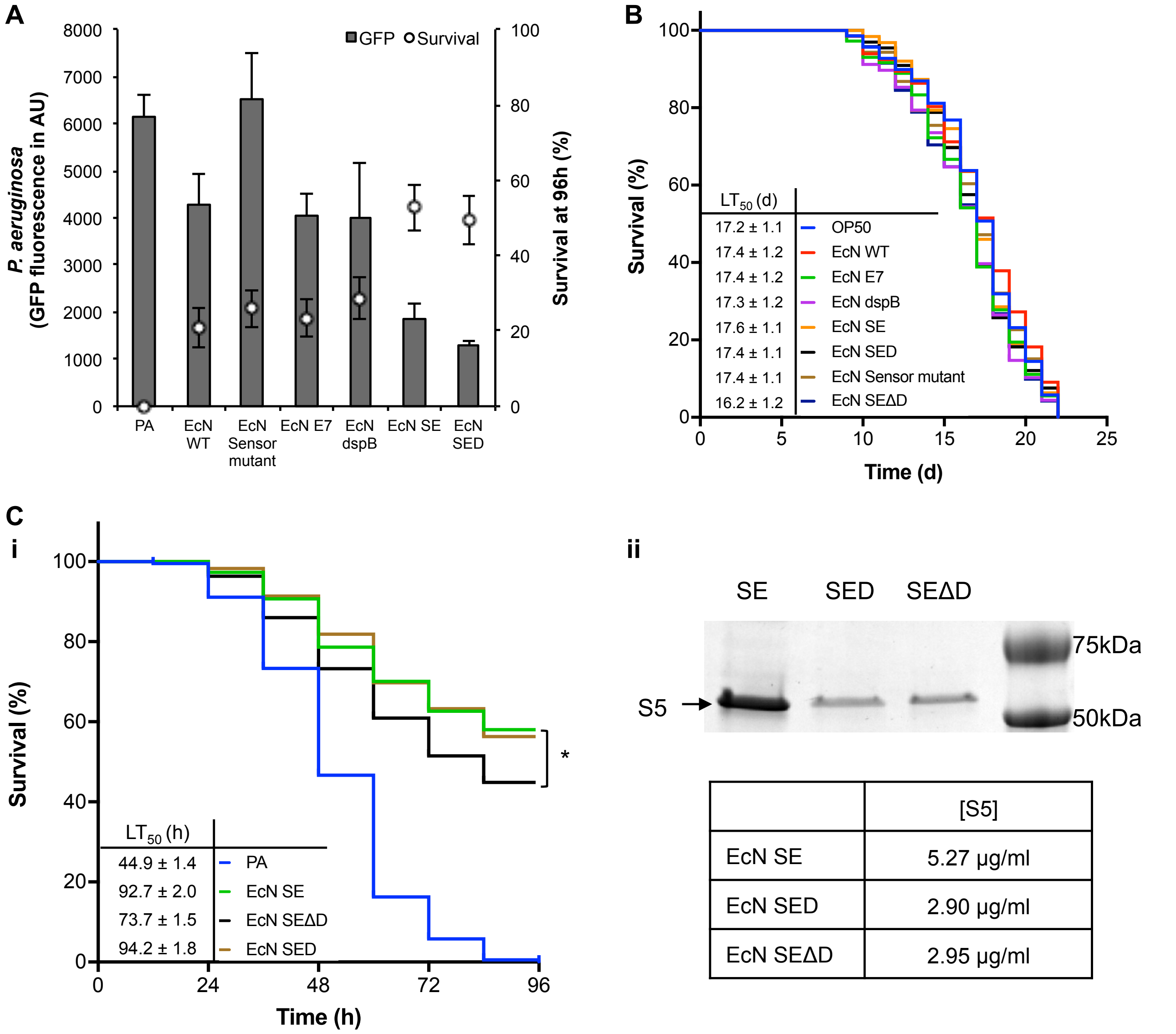

	[S5]
EcN SE	5.27 µg/ml
EcN SED	2.90 µg/ml
EcN SEΔD	2.95 µg/ml

Supplementary Figure 5: **Validation of *C. elegans* survival analysis.**

A. The pixel intensity of GFP fluorescence of the nematodes that were infected with *P. aeruginosa* (expressing GFP) followed by treatment with EcN variants, was quantified (Image J) and mean from two experiments is plotted in comparison to survival rate at 96h (n = 15-20). **B.** The survival of *C. elegans* on EcN variants alone was assessed for any detrimental effects of EcN cells on the nematodes where mean from two experiments is shown (n = 60-80). Inset table lists LT_{50} for each treatment group. **C.** (i) *C. elegans* were infected with *P. aeruginosa* for 24h and subsequently given EcN variants expressing S5 (SE), S5 with dspB (SED), or S5 with non-functional dspB (EcN SEΔD) and statistical significance was determined by Mantel-Haenszel log-rank test followed by

Bonferroni's correction. (* $P < 0.001$). Inset table lists LT_{50} for each treatment group. (ii) SDS-PAGE of purified His-tagged S5 protein sample from the extracellular supernatant of induced EcN SE, SED and SEΔD at 8h. Purification of His-tagged S5 was achieved using nickel affinity columns, and the protein was washed with 50 mM imidazole and eluted with 500 mM imidazole in PBS with 10% glycerol. The eluate was concentrated by ultrafiltration using a molecular mass cut-off membrane (Amicon Ultra-15 Centrifugal Filter Unit, Millipore). The concentration of His-tagged S5 was quantified using the standard Bradford assay.

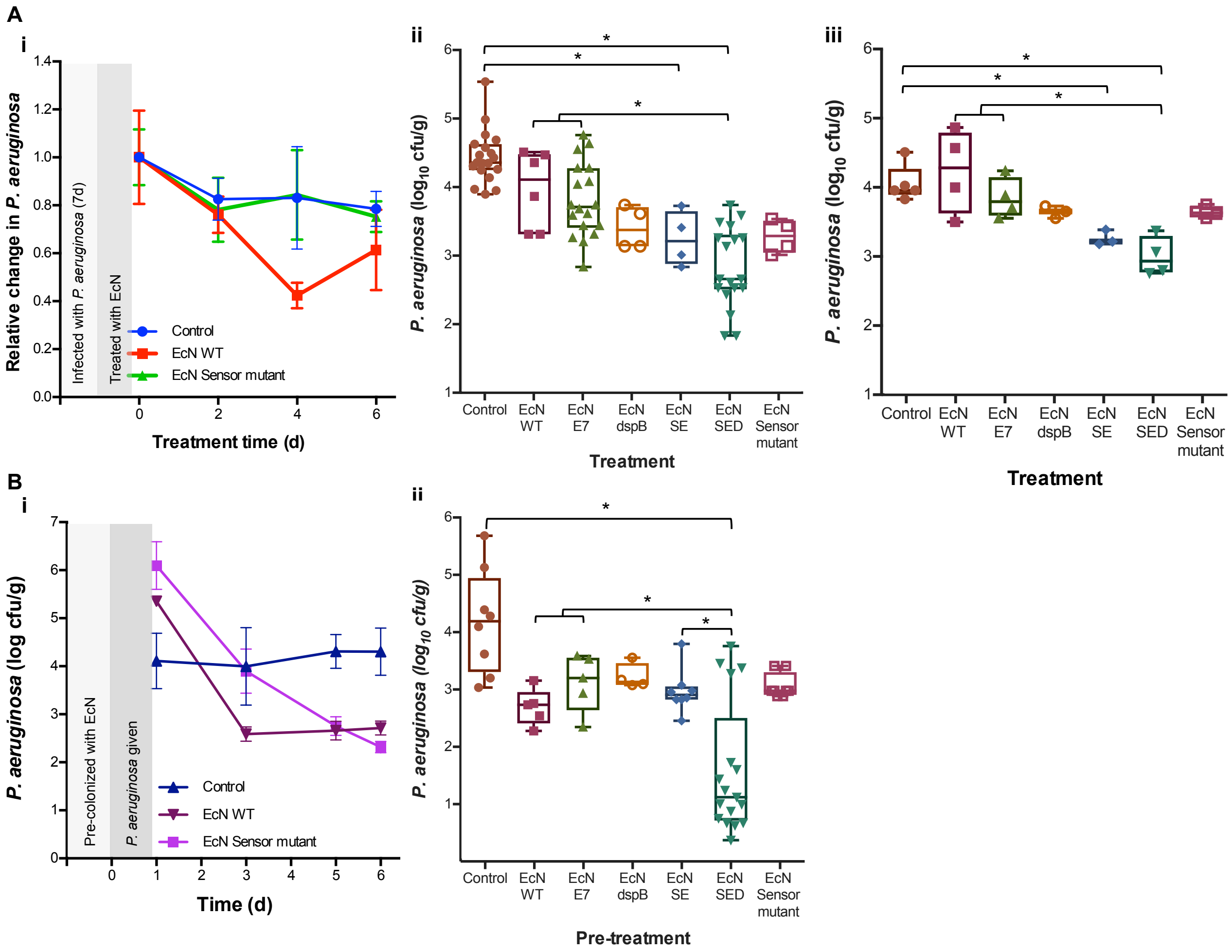

Supplementary Figure 6: **Supporting results for murine infection model.**

A. (i) Mice infected with *P. aeruginosa* for 7 days have been given EcN non-lysis control groups (wild-type and Sensor mutant) and *P. aeruginosa* was counted from feces and its relative count to the infection control group is graphed. Total viable *P. aeruginosa* cell count from (ii) fecal and (iii) colon samples of all treatment groups at day 6 post-treatment was quantified. Data from two independent experiments are shown. *Denotes statistical significance evaluated by one-way ANOVA test with Bonferroni correction ($p < 0.008$). **B.** Mice were pretreated with EcN control groups or the engineered EcN SED and subsequently infected with 10^{10}cfu of the pathogen. (i) *P. aeruginosa* cell count from non-lysis EcN (wild-type and Sensor mutant) pretreatment groups from feces and its relative count to the infection control group is graphed. (ii) Total viable *P. aeruginosa* cell count from fecal samples of all pretreatment groups at day 6 post-infection was quantified. Data

from two independent experiments are shown. *Denotes statistical significance evaluated by one-way ANOVA test with Bonferroni correction ($p < 0.008$).

Supplementary Reference

1. Lee, T. et al. BglBrick vectors and datasheets: A synthetic biology platform for gene expression. *Journal of Biological Engineering* **5**, 12-12 (2011).
